# Characterization of neocentromeric marker chromosome derived from chromosome 11: a rare entity in four patients with acute leukemia

**DOI:** 10.1007/s10577-026-09798-2

**Published:** 2026-03-26

**Authors:** Iveta Mendlikova, Jana Brezinova, Karla Svobodova, Lenka Pavlistova, Marie Valerianova, Jan Valka, Marketa Markova Stastna, Anna Jonasova, Zuzana Zemanova, Sarka Ransdorfova

**Affiliations:** 1 Institute of Hematology and Blood Transfusion, Prague, Czech Republic; 2 Center of Oncocytogenomics, Institute of Medical Biochemistry and Laboratory Diagnostics, General University Hospital and First Faculty of Medicine, Charles University in Prague, Staré Město, Czech Republic; 3 First Internal Clinic - Department of Hematology, General University Hospital and First Faculty of Medicine, Charles University in Prague, Staré Město, Czech Republic

**Keywords:** Centromere, Neocentromere, Chromosome 11, Genomic instability, Complex karyotype, Acute leukemia, Fluorescence *in situ* hybridization

## Abstract

Neocentromeres are newly formed chromosomal regions that can replace the function of traditional centromeres and are well documented in human clinical studies. However, their occurrence in neoplasia, including acute leukemia, appears to be rare. We analyzed complex karyotypes in bone marrow cells from 113 patients with acute myeloid leukemia and one patient with acute lymphoblastic leukemia using centromeric/multicentromeric fluorescence in situ hybridization and identified four cases (3.5%) with derivative chromosomes exhibiting newly formed constrictions. Three of these patients had secondary leukemia following preexisting hematological disorders, suggesting a potential role for neocentromeres in disease progression. In all four cases, neocentromeres were detected on derivative chromosome 11. To our knowledge, this is the first report of neocentromeres derived from this chromosome in acute leukemia. All four patients in our study died; however, all exhibited complex karyotypes, which are independently associated with poor prognosis and an aggressive disease course. Neocentromeres are a rare but potentially important source of genomic instability in malignant diseases. Generally, the formation of a new constriction allows mitotic rescue of acentric chromosomes, preventing their loss. An increase in genomic alterations in tumor cells predicts a more aggressive disease course and adverse outcomes. Due to limited data, the prognostic significance of neocentromeres remains unclear. Further rigorous investigation is required to deepen our understanding of the mechanisms underlying neocentromere formation and their implications in cancer.

## Introduction

Genomic instability is a hallmark of cancer and results from a wide spectrum of chromosomal and gene-level alterations that collectively drive malignant initiation, progression, and the development of therapeutic resistance (Chen et al. 2025). Hematological malignancies, in particular, illustrate the pivotal role of chromosomal pathology in cancer biology, as malignant hematologic cells frequently exhibit structural rearrangements, numerical abnormalities, and highly complex karyotypes. These alterations participate in clonal architecture and provide critical insights into the mechanisms underlying tumor heterogeneity (Oliveira et al. 2021). Thus, comprehensive cytogenetic and molecular analyses remain necessary for accurate disease classification, prognostic assessment, and elucidation of the pathways that support clonal survival and genomic diversification.

Among the key chromosomal structures essential for maintaining genomic integrity, chromosome segregation, and the transmission of genetic information are centromeres. Human centromeres are defined epigenetically and comprise large arrays of α-satellite DNA that form the primary constrictions visible in metaphase chromosomes (Willard 1991; Marshall et al. 2008). Disruption of centromere function can result in aneuploidy, mis-segregation, dicentric chromosomes, or acentric fragments. While most acentric fragments are lost during cell division, in rare cases they may be rescued through the formation of a new centromere (Amor and Choo 2002; Barra and Fachinetti 2018; Karami Fath et al. 2024). These neocentromeres form at non-centromeric chromosomal regions, lacking canonical α-satellite sequences. They are capable of forming a primary constriction, assembling a functional kinetochore, performing the role of a normal centromere and stabilize otherwise unstable chromosomal fragments (Marshall et al. 2008; Amor and Choo 2002; Barra and Fachinetti 2018; Karami Fath et al. 2024).

More than 90 constitutional neocentromeres have been described across almost all human chromosomes, revealing that no specific DNA sequence motif is required for neocentromere specification (Marshall et al. 2008; Amor and Choo 2002; Warburton 2004). Although several candidate chromosomal hotspots have been proposed, the principles of centromere repositioning and neocentromere formation remain understood.

In neoplastic settings, neocentromeres appear exceedingly rare, but their true frequency may be underestimated due to technical limitations of routine cytogenetic analysis, which may fail to detect neocentromere, particularly in complex karyotypes (Marshall et al. 2008; Barra and Fachinetti 2018; Karami Fath et al. 2024).

To date, reports document neocentromeres in selected solid tumors, including lipomatous neoplasms and lung carcinoma, and in a limited number of hematological malignancies, primarily acute myeloid leukemia and T-cell non-Hodgkin lymphoma (Blom et al. 2010; Italiano et al. 2006, 2009; Abeliovich et al. 1996; Gisselsson et al. 1999; Batanian et al. 2006; Figueiredo et al. 2009; L’Abbate et al. 2018).

The emergence of a neocentromere within a cancer clone suggests a potential mechanism by which cancer cells may bypass conventional chromosomal segregation control pathways, contributing to genomic instability and complexity of karyotypes (Barra and Fachinetti 2018; Burrack and Berman 2012). This may drive clonal evolution, uncontrolled tumor growth, and ultimately resistance to treatment (Karami Fath et al. 2024; Nassar et al. 2023). Despite this potential biological and clinical relevance, neocentromere formation in hematological malignancies remains insufficiently explored, with only isolated cases reported and with almost no data addressing their behavior in the context of complex chromosomal rearrangements.

In this study, we investigate the occurrence and characteristics of neocentromeric marker chromosomes within complex karyotypes of bone marrow cells from patients with acute leukemia. We describe four neocentromeric marker chromosomes derived from chromosome 11, representing—to our knowledge—the first reported cases of chromosome 11-associated neocentromeres in acute leukemia.

## Materials and methods

### Patients and samples

Between 2006 and 2016, bone marrow samples from 607 adult patients with newly diagnosed acute myeloid leukemia (AML), excluding those with the *PML*::*RARA* fusion, were analyzed using conventional cytogenetics. Among them, 114 patients had complex karyotypes (≥ 3 chromosomal abnormalities). All these cases were examined for the presence of monosomies by FISH using centromeric probes (Sarova et al. 2018).

However, we identified four patients with marker chromosomes that showed no detectable centromere, and these cases are presented here. In one patient, the initial diagnosis was later revised to pre-B acute lymphoblastic leukemia with excess blasts. Given the cytogenetic findings and the rarity of neocentromeres, this case was retained and is presented as Patient No. 3.

### Conventional cytogenetics

Standard G-banding (Wright staining) was carried out, and metaphases were analyzed using the IKAROS imaging system (MetaSystems, Altlussheim, Germany). Karyotypic findings were interpreted and reported according to the International System for Human Cytogenomic Nomenclature (ISCN), with 20 metaphases analyzed in most cases, as applicable (McGowan-Jordan et al. 2024).

### Fluorescence *in situ* hybridization (FISH)

Complex karyotypes of all patients were characterized using multicolor FISH (mFISH) (24XCyte Human Multicolor FISH Probe Kit) and/or multicolor banding (mBAND) (XCyte color kits) and analyzed using the ISIS imaging system (MetaSystems).

All patients were tested for chromosomal presence using centromere specific FISH with the XCyting Centromere Multi-Color Probe Mix (cen-mFISH, MetaSystems), Vysis chromosome enumeration probes (CEP) (Abbott, Des Plaines, IL) and Satellite Enumeration (SE) probes (Kreatech Diagnostics, Amsterdam, the Netherlands). The cen‑mFISH probe set is capable of detecting 18 chromosome pairs (1–12, 15–19 and X). In cases involving rearrangements of chromosomes not covered by the cen‑mFISH set, we used individual centromeric probes—CEP20 (Abbott Vysis) and SE 13/21 and SE 14/22 (Kreatech). If we had doubts about probe signal by cen-mFISH, we confirmed it by individual centromeric probes. The cut-off thresholds for monosomy and trisomy detection were set at 5% and 2.5%, respectively.

For chromosome 11, Vysis *MLL* Break Apart Rearrangement probe (11q23.3) and TelVysion 11q probe were additionally used (Abbott Vysis).

All procedures were performed according to the manufacturers’ protocols.

### CGH–SNP array

Some patients (for whom sufficient material was available) were examined by CGH–SNP Array. DNA was extracted from fixed bone marrow cells using the QIAamp DNA Mini Kit (Qiagen, Germantown, MD, USA). Sex-matched reference DNA from the NA12878 cell line (Coriell Institute, Camden, NJ, USA) was used. Hybridization was performed using the CytoChip Cancer SNP 180 K platform (BlueGnome, Illumina, San Diego, CA), which was scanned with the Agilent G2565CA Microarray Scanner and analyzed using BlueFuse Multi v3.1–4.1 software (BlueGnome, Illumina).

## Results

**Fig. 1:**
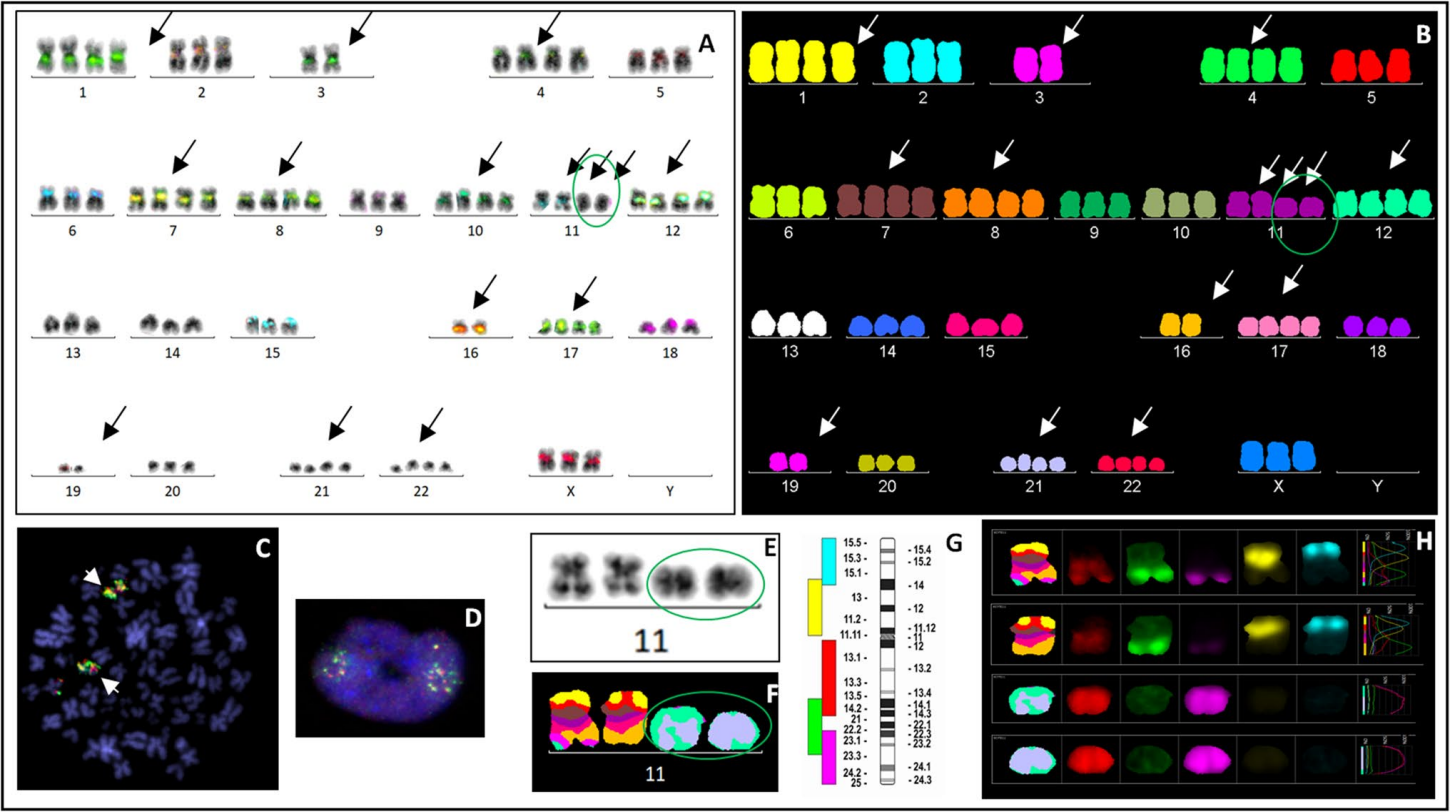
Patient 1: A near-triploid karyotype including two derivative chromosomes 11 was demonstrated by mFISH (**B**). cen-mFISH showed no signal for the chromosome 11 centromere on derivative chromosomes 11 (**A**). FISH with the break-apart LSI MLL probe (11q23) showed *KMT2A* gene amplification in a metaphase (**C**) and in an interphase cell (**D**). G-banding identified two normal and two derivative chromosomes 11 (**E**). mBAND XCyte 11 demonstrated one normal chromosome 11, one chromosome with a deletion del(11)(q23.3), and two marker chromosomes containing exclusively 11q23–qter material with its amplification (**F, H**). A labelling scheme for the mBAND probe for chromosome 11 is shown in (**G**). Neocentric chromosomes are highlighted with green ovals

**Fig. 2:**
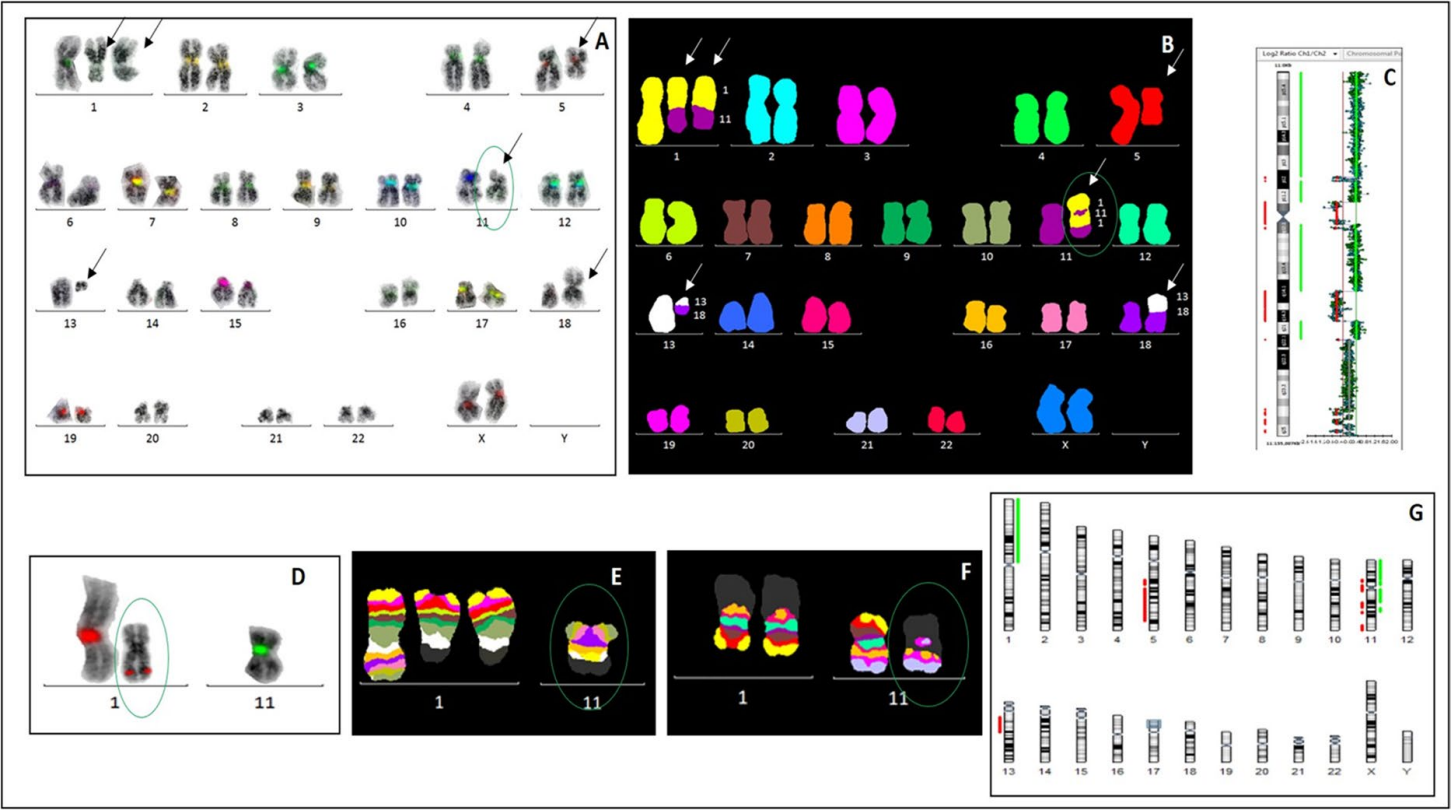
Patient 2: A complex karyotype was demonstrated by mFISH (**B**) and aCGH/SNP analysis (**G**). No classical centromere signal on the marker chromosome (green oval) was detected by cen-mFISH (**A**) and FISH with individual centromeric probes for chromosomes 1 and 11 — CEP1 (SpectrumOrange)/CEP11 (SpectrumGreen) (**D**). Partial aCGH/SNP profiling of chromosome 11 confirmed the loss of the centromeric 11p11.2–11q12.1 region and copy-number alterations affecting other chromosome 11 segments (**C**). The structure of the neomarker and the insertion of the 11q23 region into chromosome 1 material were identified using mBAND XCyte 1 (**E**) and mBAND XCyte 11 (**F**)

**Fig. 3:**
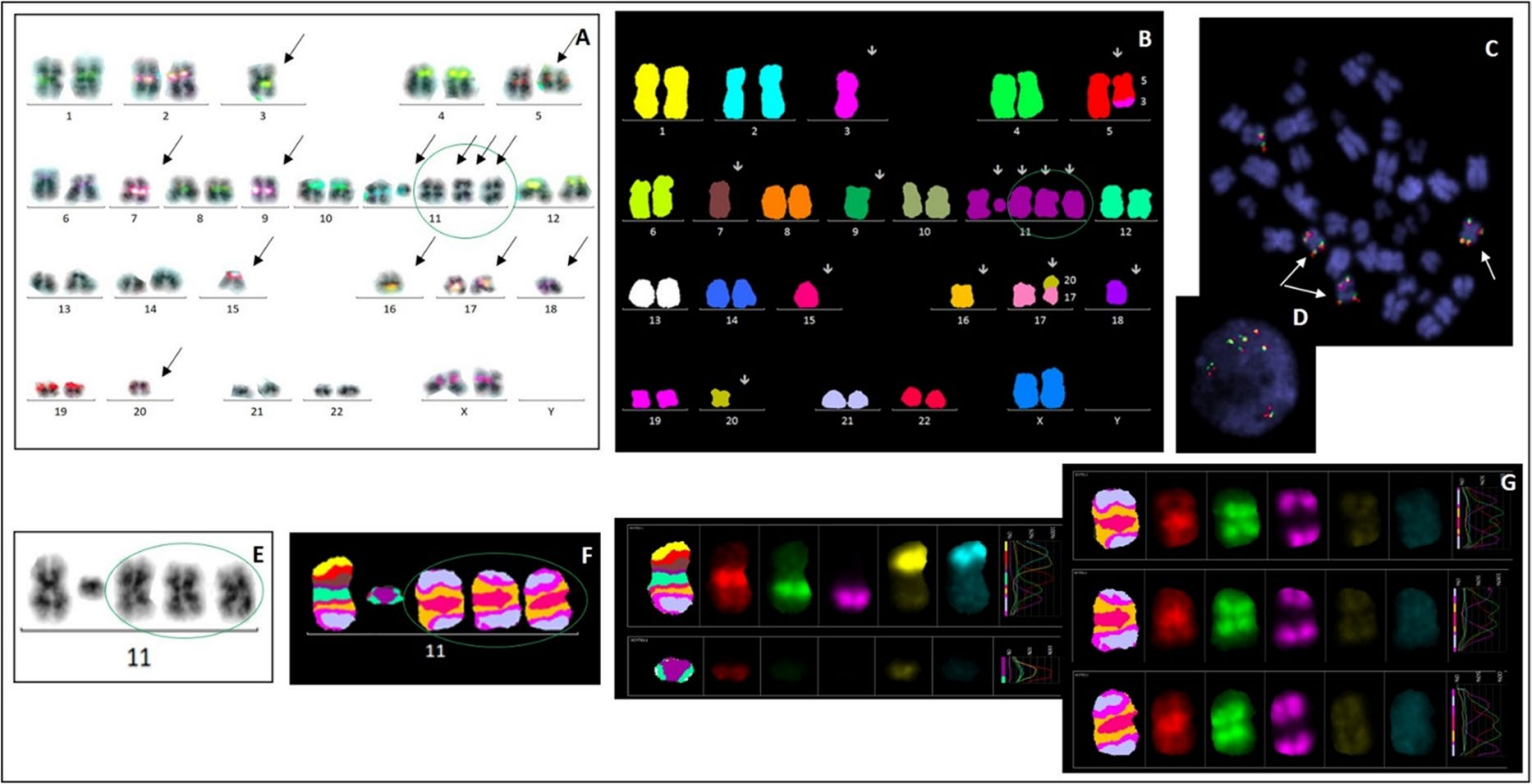
Patient 3: A complex karyotype was revealed by mFISH (**B**). No centromere 11 signal was detected by cen-mFISH (**A**). An inverted duplication of chromosome 11 was demonstrated by the mirror localization of the *KMT2A* (MLL) gene on both arms of the chromosome using the break-apart LSI MLL probe (11q23) in a metaphase (**C**) and in an interphase cell (**D**). G-banding identified one deleted chromosome 11 and three copies of inv dup(11) (**E**). The involved chromosomal bands in inv dup(11)(q13qter) were further defined by mBAND XCyte 11 (**F, G**). Neocentric chromosomes are highlighted with green ovals

**Fig. 4:**
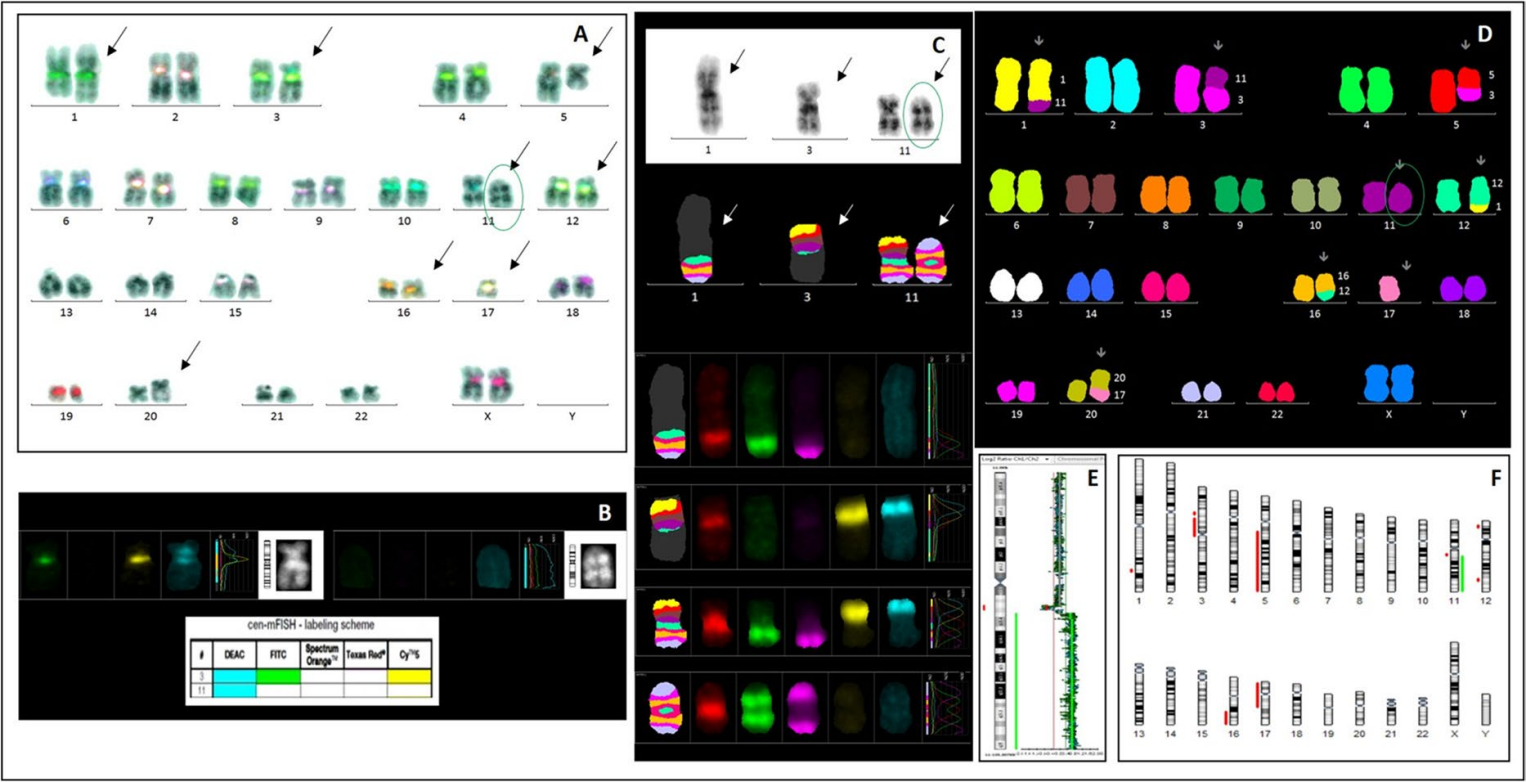
Patient 4: A complex karyotype was demonstrated by mFISH and aCGH/SNP (**D, F**). Presence of the centromeres of chromosome 3 (combination of blue, green, and yellow) and chromosome 11 (blue only) in dic(3;11), and absence of a centromere signal on inv dup(11), were detected by cen-mFISH (**A, B**). The chromosomal 11 bands involved in t(1;11) — 11q13.1, in dic(3;11) — 11q13.1, and in the inverted duplication — 11q13.3qter, were identified using mBAND XCyte 11 (**C**). aCGH/SNP analysis confirmed loss of the 11q13.1–11q13.3 region and duplication of 11q13.3–qter (**E**). The neocentric chromosome is highlighted with a green oval

Neocentromeric marker chromosomes were identified using an integrated cytogenetic and genomic approach. Conventional G-banded karyotyping was performed for initial detection of structural and numerical chromosomal variations for identify complex and marker chromosomes. Centromere-specific FISH using α-satellite probes was applied to confirm the absence of canonical centromeric sequences. Chromosomal origin and structural rearrangements were characterized using multicolor FISH (m-FISH) and multicolor banding (mBAND). High-resolution SNP array analysis was subsequently used to define copy number alterations and refine breakpoint mapping, enabling comprehensive characterization of neocentromeric marker formation. Neocentromeric markers were identified in the complex karyotypes of three out of 113 patients with AML included in the study (2.7%) and in one patient whose diagnosis was later revised to pre-B acute lymphoblastic leukemia with excess blasts (Table 1; Figs. 1, 2, 3, 4). The karyotypes of the remaining AML patients without complex karyotypes were established without any uncertainty regarding the presence of neocentromeres.

**Table 1:** Summary of the clinical and cytogenetic data of patients with neocentromeric marker

No	Sex/Age	Diagnosis	Karyotype of malignant clone (conventional cytogenetics/mFISH)	Neocentromere localization (mBAND)
1	F/65	sAML	74 ~ 77 < 3n >,XXX, + 1,−3, + 4, + 7, + 8, + 10,del(11)(q23.3),** + der(11)(?) × 1 ~ 2**, + 12,−16, + 17,−19, + 21, + 22[cp11]	amp(11)(q23.3q25)—not specified
2	F/64	sAML	46 ~ 48,X,-X,t(X;5)(q13;q12), + 1,der(1)t(1;11)(?;?) × 2,del(5)(q13q33),del(11)(q11),t(13;18)(q12;p11),−13,−18,**der(?)t(1;11)(?;?)**, + 1 ~ 3mar[cp22]	neo(11)(1qter → 1q31::11q23.3 → neo → 11q23.3::1q31 → 1q12::11q22.2 → 11qter)
3	F/68	pre-B-ALL	37 ~ 42,XX,−3,der(5)t(3;5)(?;q22),−7,−9,der(11)del(11)(p11)del(11)(q12),** + inv dup(11)(q13.3qter) × 3**,−15,−16,dic(17;20)(p11.2;q11.2),−18[cp16]	neo(11)(11qter → 11q13.3 → neo → 11q13.3 → 11qter)
4	F/76	sAML	45,XX,der(1)t(1;11)(q32;q13.1),dic(3;11)(p11;q13.1),der(5)t(3;5)(p11;q12),der(12)t(1;12)(q32;q24),der(16)t(12;16)(q24;q12) [8]/45,idem,** + inv dup(11)(q13.3qter)**,−17, der(20)t(17;20)(q12;q11) [13]	neo(11)(11qter → 11q25 → neo → 11q25 → 11q13.3::11q13.3 → 11qter)

Abbreviations: *F* female, *sAML* secondary acute myeloid leukemia, *pre-B-ALL* pre-B acute lymphoblastic leukemia

### Patient 1

A 65-year-old woman with a history of follicular lymphoma (FL) was diagnosed with secondary AML with dysplasia in October 2011. Cytogenetic analysis revealed a near-triploid complex karyotype with gains and losses of multiple whole chromosomes (Table 1; Fig. 1A, B).

However, the marker chromosome with neocentric formation for chromosome 11 was the only structural change that was identified with one to two copies of a derivative chromosome 11 fragments with 11q23-qter amplification as detected by cen-mFISH and mFISH (Fig. 1A, E–H). FISH demonstrated multiple copies of the *KMT2A* (*MLL*) gene and the 11q subtelomeric region, while no centromeric α-satellite signal for chromosome 11 was detected on the derivative chromosome (Fig. 1A, C, D). mBAND confirmed high-level amplification of the 11q23.3–q25 region (Fig. 1F–H).

The patient died in February 2012 due to aggressive disease with persistent complex cytogenetic abnormalities.

### Patient 2

A 64-year-old woman was diagnosed with myelodysplastic syndrome (MDS RAEB-2) in October 2013. Two cytogenetic clones were identified: one with isolated del(5)(q13q33) and a dominant clone with a complex karyotype including del(5)(q13q33) and other rearrangements of chromosomes 1, 11, 13, and 18 (Table 1; Fig. 2B).

mBAND detected a neocentric chromosome, neo(11), composed of rearranged segments from chromosomes 1 and 11. The constriction of neo(11) corresponded to an inserted 11q23 fragment within chromosome 1 material (Table 1; Fig. 2E, F). FISH revealed weak chromosome 1 centromeric signals outside the constriction and an absence of chromosome 11 centromeric signal on neo(11) (Fig. 2A, D). CGH-SNP array analysis confirmed loss of the 11p11.2–11q12.1 region and other copy number alterations (Fig. 2C, G).

By January 2014, the disease had progressed to unspecified AML. The complex clone persisted, with additional monosomies of chromosomes 13 and 18 and one to three newly emerging supernumerary marker chromosomes. The patient died from complications related to the disease in September 2014.

### Patient 3

A 68-year-old woman was diagnosed with acute leukemia in February 2014, later classified as pre-B acute lymphoblastic leukemia with excess blasts. mFISH revealed a complex karyotype with multiple numerical and structural abnormalities including loss of chromosomes 3, 7, 9, 15, 16, and 18; a dicentric chromosome dic(17;20); and additional rearrangements of chromosomes 3, 5, and 11 (Table 1; Fig. 3B).

mBAND analysis for chromosome 11 identified three copies of inv dup(11)(q13.3qter) (Fig. 3F, G). FISH confirmed mirrored localization of the *KMT2A* (*MLL*) gene on both arms of the inverted duplication and absence of a chromosome 11 centromeric signal (Fig. 3A, C, D). The constriction was symmetrically located within the 11q13.3 region (Fig. 3E). The patient died due to disease-related complications in March 2014.

### Patient 4

A 76-year-old woman was diagnosed with AML-M2 in January 2016 following chronic myelomonocytic leukemia. A complex karyotype was observed, involving alterations of chromosomes 1, 3, 5, 11, 12, and 16, and a dicentric chromosome dic(3;11), along with additional abnormalities including + inv dup(11)(q13.3qter), monosomy 17, and der(20)t(17;20)(q12;q11) in most cells (Table 1; Fig. 4D, E).

Cen-mFISH confirmed the presence of centromeres 3 and 11 on dic(3;11), monosomy 17, and absence of any centromeric signal on the inv dup(11) marker (Fig. 4A, B). mBAND and CGH-SNP array analyses demonstrated that the rearranged chromosome 11 material corresponded to inv dup(11)(q13.3qter) (Fig. 4C, E). In contrast to Patient 3, the new constriction was located at the distal end of the marker, within the 11q25 region (Fig. 4A, B, C). The patient died as a result of complications attributable to the underlying disease in February 2016.

## Discussion

Neocentromeres are well documented in constitutional human cytogenetics; however, their occurrence in cancer remains unclear. Their apparent rarity may reflect both biological constraints and technical limitations. In routine diagnostics, neocentromeres can easily escape detection, particularly in the context of complex karyotypes, unless targeted cytogenomic methods are employed.

In this study, we focused specifically on highly complex karyotypes in leukemia, where structural chromosome instability is pronounced and where neocentromere formation might be expected. Nonetheless, our findings confirm that neocentromeres remain rare even in this setting, being identified in only 3 of 113 AML patients with complex karyotype (2.7%). The reason why neocentromeres remain rare—even in markedly unstable cancer genomes—is not understood, and additional studies are required.

The chromosomal site of neocentromere formation remains an area of active investigation. Evidence from inactive centromeres in dicentric chromosomes, as well as the widespread distribution of neocentromeres, strongly suggests that epigenetic mechanisms predominate over DNA sequence specificity. Although certain AT-rich regions or duplication-rich genomic intervals may provide favorable environments for neocentromere establishment (Marshall et al. 2008).

To date, neocentromeres in hematologic malignancies have been described on chromosomes 1, 3, 8, 10, and 12 (Abeliovich et al. 1996; Gisselsson et al. 1999; Batanian et al. 2006; Figueiredo et al. 2009; L’Abbate et al. 2018). Our cases represent, to our knowledge, the first report of neocentromeres arising from chromosome 11 in acute leukemia. In constitutional settings, neocentromeres have been identified on almost all human chromosomes, confirming that no specific DNA sequence motif is required for neocentromere specification. However, several neocentromere hotspots have been reported 3q, 8p, 9p, 13q, 15q, and Yq (Marshall et al. 2008; Amor and Choo 2002; Warburton 2004). The frequent association of neocentromeres with these particular chromosomal regions may reflect the incompatibility of marker chromosomes derived from other loci with embryonic survival and development. In hematologic malignancies, neocentromeres tend to arise on chromosomes that are commonly affected by structural abnormalities in cancer in general, such as chromosomes 1, 3, 8, 10, 11, and 12.

Neocentromere formation may also depend on the type and extent of the underlying chromosomal alteration. Inverted duplications represent the most common architecture associated with neocentromeres (Marshall et al. 2008). Two of the markers in our cohort originated from inverted duplications, inv dup(11)(q13.3qter). These inverted duplications typically display a single functional constriction despite their symmetrically duplicated arms. Depending on the precise location of the new constriction, the marker can appear symmetrical or asymmetrical (Marshall et al. 2008). In our study, Patient 3 exhibited a central constriction at 11q13.3, whereas Patient 4 showed a distal constriction at 11q25. Distally localized neocentromeres have been described in other AML and T-cell non-Hodgkin lymphoma cases, and they seem to be more common than symmetrical inverted duplications in hematologic malignancies. The underlying reason remains unclear.

Inverted duplications often result in partial trisomy or tetrasomy (Marshall et al. 2008). In our patients this mechanism produced substantial copy-number gains within 11q. Patient 4 exhibited partial tetrasomy of 11q13.3–qter, while Patient 3 had penta- to heptasomy of the same region. Such rearrangements may lead to increased gene copy number and activation of oncogenes. In both cases, the *KMT2A* gene (11q23.3), which is frequently altered in patients with acute leukemia, is located within the amplified segment (Poppe et al. 2004; Braekeleer et al. 2005). Thus, neocentromere formation may indirectly contribute to leukemogenesis by permitting the clonal stabilization of rearrangements that increase oncogene dosage.

Other mechanisms of neocentromere formation include linear or circular marker chromosomes arising after interstitial deletions (Marshall et al. 2008; Gisselsson et al. 1999; L’Abbate et al. 2018). In Patient 1, the derivative chromosome resulted from amplification of 11q23–q25, although the exact site of constriction remains unclear. Notably, this amplification involved the proto-oncogene *KMT2A*. In Patient 2, a neocentromere was detected in the 11q23.3 region, inserted into rearranged and fused chromosomes 1 and 11.

The frequent co-occurrence of neocentromeres with additional abnormalities supports the hypothesis that neocentromere formation in acute leukemia is most likely a secondary event reflecting disease progression (Abeliovich et al. 1996; Gisselsson et al. 1999; Batanian et al. 2006; Figueiredo et al. 2009; L’Abbate et al. 2018). All four patients in this study presented with complex karyotypes, a finding observed in 10–12% of AML cases. In general, the presence of complex karyotypes is associated with older age, advanced disease, and poor prognosis (Schoch et al. 2005; Mrózek 2008). Three of the four patients had secondary-type leukemia with a history of previous hematologic disease, suggesting that neocentromere formation emerges during disease evolution rather than at disease initiation. However, the specific impact of chromosome 11 neocentromere formation remains unresolved.

The formation of a functional constriction allows mitotic rescue and stabilization of acentric fragments generated by chromosomal rearrangements. In our cases, the structurally compromised chromosomes 11 were likely retained through the acquisition of neocentromere activity, which may have restored effective centromere function, facilitated mitotic segregation, and enabled continued clonal expansion despite underlying structural instability. Such events contribute to genomic instability, clonal evolution, and potentially adverse clinical outcomes.

All four patients in our cohort demonstrated poor overall survival, likely reflecting the presence of highly complex karyotypes and advanced genomic instability. The probable involvement of neocentromere-mediated mechanisms may have further contributed to disease progression by enabling clonal persistence of structurally abnormal chromosomes. However, the specific role of chromosome 11 neocentromere formation remains unresolved and warrants further systematic investigation.

In conclusion, neocentromeres are rare but potentially biologically significant contributors to genomic instability in hematologic malignancies. Their detection requires targeted cytogenomic techniques and their presence appears to coincide with other complex rearrangements, supporting a secondary origin. Neocentromeres may facilitate amplification of oncogenes, promote mitotic rescue, and enable clonal persistence of structurally abnormal chromosomes. However, their prognostic significance remains uncertain. The data suggest possible biological relevance of neocentromere-mediated survival of structurally abnormal chromosomes, but no definitive conclusions can be drawn regarding clinical impact. Further studies with larger cohorts are necessary to clarify the role of neocentromere formation, particularly on chromosome 11, in hematological malignancies.

## Data Availability

Data available upon request.
